# Occipital Condyle Syndrome As the Initial Presentation of Recurrence of Metastatic Breast Cancer: A Case Report

**DOI:** 10.7759/cureus.34567

**Published:** 2023-02-02

**Authors:** Sowbharnika Arivazhagan, Guru Prasad Parthiban, Vishal Busa, Catalina Negulescu

**Affiliations:** 1 Internal Medicine, Baton Rouge General, Baton Rouge, USA

**Keywords:** palliative care, metastasis, breast cancer, skull base metastasis, occipital condyle syndrome

## Abstract

Skull-base metastasis is extremely rare. Various syndromes have been identified based on the anatomical involvement of the metastatic tumor. Occipital condyle syndrome (OCS) occurs with involvement of occipital bone and compression of the hypoglossal canal. OCS is very rare and usually has an underlying widely disseminated metastatic cancer. We present a 66-year-old female who initially presented with tongue deviation and occipital headache. MRI revealed a mass compressing the occipital bone and hypoglossal canal. Further work-up revealed metastatic breast cancer.

## Introduction

Metastasis to the skull is uncommon and accounts for only 4% of cancer-related metastasis, and their incidence is further increased with the advent of current advanced imaging modalities [1,2]. Breast cancer is the second most common cause of metastasis to the brain. Metastasis in the brain and other distant organs poses a significant challenge in managing breast cancer [2,3]. Even though brain metastasis is common in breast cancer, skull base metastasis is comparatively rare and commonly present as cranial neuropathies with widely varying presenting symptoms, making it difficult to diagnose. Breast cancer is followed by lung and prostate cancers in metastasis to the skull base [4,5]. Other rare malignancies include lymphoma, colon, renal, thyroid, and melanoma [4]. Based on the involvement of cranial nerves (either single or multiple) and the location of the skull base, these were divided into a spectrum of syndromes like orbital syndrome, cavernous sinus syndrome, middle fossa syndrome, jugular foramen syndrome, numb chin syndrome and occipital condyle syndrome [5]. In this report, we discuss a patient with triple-negative breast cancer who presented with cranial nerve deficits and was later found to have metastatic disease to the occipital condyle. We emphasize the importance of recognizing occipital condyle syndrome promptly, which is rare and fatal if undiagnosed.

## Case presentation

**Figure 1:**
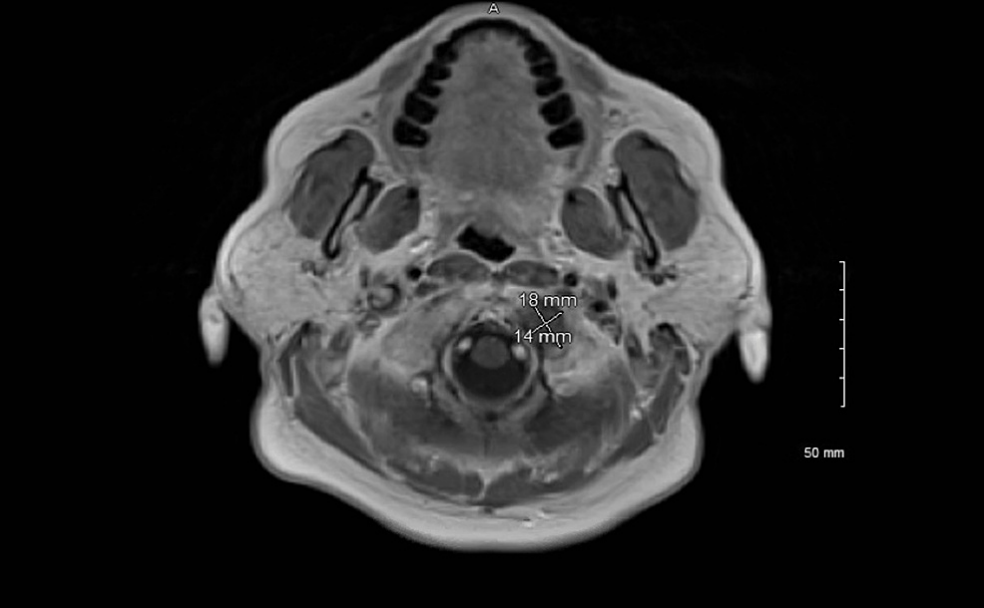
Mass in the occipital condyle

The patient is a 66-year-old female with a history of triple-negative breast cancer (post-chemotherapy in remission) who presented to the Emergency Department with chief complaints of headaches, hoarseness of voice, and tongue deviation. Patient reported that her tongue remained deviated to the left for three days and had been having difficulty with swallowing. She also had been having occipital headaches with radiation to the left temporal region for three weeks. Headaches were sharp in nature and persistent, with no specific aggravating or relieving factors. Symptoms were also associated with hoarseness with hypophonic voice, cough, and dry throat. The rest of the review of the system was unremarkable. The patient’s past medical history was significant for triple-negative breast cancer diagnosed 15 years ago (Primary 1.4 cm, 1/15 nodes positive), has received chemotherapy, and has been in remission since. Vitals were stable. Physical examination was remarkable for hypophonic voice, tongue deviation to the left, diminished gag reflex, decreased palatal movement on the left, and mild left-sided tongue fasciculations. Initial imaging with computed tomography (CT) of the head did not reveal any abnormality. Given cranial nerve X and XII involvement, an MRI head was obtained, which was impressive for soft tissue mass within the left occipital bone with involvement of the left hypoglossal canal (Figure 1). This was consistent with occipital condyle syndrome (OCS), explaining the patient's headache, tongue deviation, and hypotonic voice. Due to suspected metastatic disease, CT of the chest and abdomen was ordered, showing widespread metastatic disease in the lung, liver, right adrenal, L4 vertebrae, and distal pancreatic body. CT-guided biopsy of an accessible right hepatic mass was performed with pathology revealing metastatic breast cancer, ER-negative, and insufficient sample for PR/HER2 testing. Due to the severity of her headaches, radiation therapy to the mass of the base of the skull was recommended. She received two out of five radiation therapies while in the hospital, with significant improvement in her headaches. She was later discharged to follow up with an oncologist and radiation oncologist as an outpatient. 

## Discussion

OCS is a rare entity comprising unilateral occipital headache with radiation to the mastoid, vertex, and ear and may be associated with scalp tenderness [3,4]. It is often accompanied by ipsilateral hypoglossal palsy [4]. OCS is most commonly a result of skull-based metastasis. Among the five clinical syndromes associated with skull-based metastasis (the orbital, para sellar, middle fossa, jugular foramen, and occipital condyle syndromes), OCS is very rare and often is misdiagnosed due to a lack of awareness of its clinical presentation [5]. Breast and prostate cancers are the most common primary tumors in women and men to metastasize to the occipital condyle respectively [6]. 

In OCS, the 12th nerve is entrapped as it exits the skull through a hypoglossal canal or anterior condylar foramina resulting in ipsilateral paresis of the hypoglossal nerve [7]. This results in ipsilateral tongue deviation with atrophy and fasciculations. Symptoms may be associated with dysarthria and dysphagia (usually secondary to tongue palsy) [6]. OCS may cause convergence between trigeminal and various cervical efferents, which results in occipital pain with radiation, as mentioned above [4]. The extension of the mass may also result in IX, X, and XI cranial nerve palsies [4]. Hematogenous dissemination is the most common route of spread. Water-soluble chemotherapeutic agents cannot cross the blood-brain barrier. Hence, micrometastasis can remain clinically silent for years and manifest later with neurological impairment and without any other evidence of systemic disease [4]. Usually, OCS presents as a late event in the cancer progression and carries a very poor prognosis [6]. In an old series of 100 patients with hypoglossal nerve palsy from 1996, half of them with non-traumatic palsy had underlying malignancy [8].

MRI with gadolinium enhancement remains the most sensitive and specific diagnostic tool in diagnosing OCS [7]. MRI detects both soft tissue infiltration by the tumor as well as soft tissue infiltration [6]. Treatment of OCS includes corticoid therapy, radiotherapy, or a combination of both, mainly as a palliative approach to alleviate pain [6]. In patients with sensitive tumors such as breast and prostate, chemotherapy or hormonal therapy should be considered if appropriate to prolong survival [4]. 

## Conclusions

OCS is a very rare clinical entity and symptom onset is usually very late during cancer progression. OCS being the initial clinical manifestation of a metastatic disease usually carries a very poor prognosis given its wide dissemination. Prompt diagnosis both clinically and radiologically is required at the earliest to determine the further course of cancer treatment. In patients who are not candidates for systemic therapy, radiotherapy remains an excellent option for alleviation of symptoms and improving the quality of life.
